# Southern California Pediatric and Adolescent Cancer Survivorship (SC-PACS): Establishing a Multi-Institutional Childhood, Adolescent, and Young Adult Cancer Survivorship Consortium in Southern California

**DOI:** 10.7759/cureus.21981

**Published:** 2022-02-07

**Authors:** Carol Lin, Nicole Baca, Christine Yun, Saro Armenian, David R Freyer, Fataneh Majlessipour, Lisa Mueller, Dennis J Kuo, Jacqueline Casillas, Keri Zabokrtsky, Louis Ehwerhemuepha, Lilibeth Torno

**Affiliations:** 1 Department of Pediatric Oncology, Hyundai Cancer Institute, Children’s Hospital of Orange County, Orange, USA; 2 Department of Pediatrics, Samuel-Oschin Comprehensive Cancer Institute, Cedars-Sinai Medical Center, Los Angeles, USA; 3 Department of Pediatrics, City of Hope, Duarte, USA; 4 Department of Population Sciences, City of Hope, Duarte, USA; 5 Department of Pediatric Oncology, Cancer and Blood Disease Institute, Children’s Hospital Los Angeles, Los Angeles, USA; 6 Department of Pediatric Oncology, Keck School of Medicine, University of Southern California, Los Angeles, USA; 7 Division of Pediatric Hematology and Oncology, Department of Pediatrics, Kaiser Permanente Medical Center, Los Angeles, USA; 8 Division of Pediatric Hematology-Oncology, University of California San Diego, San Diego, USA; 9 Division of Pediatric Hematology-Oncology, Rady Children’s Hospital San Diego, San Diego, USA; 10 Division of Hematology and Oncology, Department of Pediatrics, University of California Los Angeles, Los Angeles, USA; 11 Department of Pediatrics, Jonathan Jaques Children’s Cancer Institute, Miller’s Children’s Hospital, Long Beach, USA; 12 Research Institute, Children’s Hospital of Orange County, Orange, USA; 13 Department of Computational Research, Children’s Hospital of Orange County, Orange, USA; 14 Department of Pediatric Oncology, Hyundai Cancer Institute, Children's Hospital of Orange County, Orange, USA

**Keywords:** survivorship needs, survivorship consortium, childhood/adolescent cancer survivors, pediatric cancer, cancer survivorship

## Abstract

Introduction

Given their risk for late effects and early mortality, childhood/adolescent cancer survivors (CACSs) should receive longitudinal monitoring and care. The Southern California Pediatric and Adolescent Cancer Survivorship (SC-PACS) consortium was established in February 2017 to combine resources and expertise across seven participating survivorship programs. Its over-arching objective is to address the unique needs of its demographically diverse CACS population through collaborative survivorship research and care initiatives. The first SC-PACS study was an assessment of survivorship needs and evaluation of current services as reported by CACSs and their parents/primary care givers (PPCGs) receiving survivorship care at consortium sites.

Methods

As an initial investigation, a cross-sectional survey for CACSs and their parents/primary care givers was conducted. The goal was to enroll 10 CACSs and 10 PPCGs from each of the seven institutions (total of 140 participants). The eligibility criteria for CACSs were age ≥13 years at the time of enrollment, >2 years from the end of treatment, sufficient cognitive function to complete the survey, and English or Spanish language proficiency. For CACSs <13 years old, their PPCGs completed the survey. This was a convenience sample using frequencies and proportions to describe participant characteristics and survey responses, which were entered into a Research Electronic Data Capture (REDCap) database.

Results

Across the consortium, of the recruitment target of 140 participants (CACSs, n=70; PPCGs, n=70), 127 (90.7%) participants were enrolled. Of the 127 participants enrolled, 65 (51.2%) were CACSs and 62 (48.8%) were PPCGs. The majority of participants were female (51.2%), were Hispanic (62.2%), spoke English as the primary language at home (57.5%), and were diagnosed between one to four years of age (45.7%). Information considered most important by both CACSs and PPCGs was related to cancer diagnosis (90.8%) and future risks as a result of cancer treatment received (98.0%). Overall, 78% of CACSs and PPCGs found the survivorship information (treatment summary) useful, and 83% felt that they received the right amount of information about their cancer.

Conclusion

Our aim was to obtain baseline data that would characterize our CACS population, inform consortium priorities, and establish a collaborative research platform. The ultimate goal of the consortium is to develop a comprehensive survivorship care approach that addresses the most important needs of cancer survivors in our catchment area and promotes best practice interventions. Future plans are to expand the needs assessment survey to obtain a wider representation of the survivor population at SC-PACS institutions, helping create strategies to improve cancer-specific education, delivery of treatment summary, and access to community resources for this demographically and socioeconomically diverse population.

## Introduction

Due to remarkable progress in the treatment of cancer among children and adolescents, their aggregate five-year survival now exceeds 84% [1-3]. However, large cohort and population-based studies have documented the high burden of morbidity and mortality associated with cancer treatment at a young age [4-10]. These studies have included mostly non-Hispanic white participants and few racial and ethnic minorities [11-14]. These underrepresented populations of survivors may have different outcomes and needs that have been understudied to date. With the increasing diversity of the United States [15], cancer survivorship programs must ensure their childhood/adolescent cancer survivors (CACSs) are appropriately represented in research studies and have access to culturally competent care. The racial, ethnic, and sociocultural diversity of southern California’s population makes it an ideal environment to gain insight into the most pressing concerns for CACSs living in this region and beyond.

Consequently, the Southern California Pediatric and Adolescent Cancer Survivorship (SC-PACS) consortium was established in February 2017 with the over-arching objective of addressing the unique needs of its demographically diverse CACS population through collaborative survivorship research and care initiatives. The demographic reach of the SC-PACS consortium encompasses Los Angeles, San Diego, and contiguous counties, a region accounting for approximately 40% of the California population.

The first SC-PACS study was an assessment of survivorship needs and evaluation of current services as reported by CACSs and their parents/primary care givers (PPCGs) receiving survivorship care at consortium sites. Our goal was to obtain baseline data that would characterize our CACS population, inform consortium priorities, and establish a collaborative research platform.

## Materials and methods

The consortium includes seven cancer treatment centers for children and adolescents, including Cedars-Sinai Medical Center (Los Angeles, CA), Children’s Hospital Los Angeles (Los Angeles, CA), Children’s Hospital of Orange County (Orange, CA), City of Hope (Duarte, CA), Kaiser Permanente Medical Center (Los Angeles, CA), Rady Children’s Hospital San Diego (San Diego, CA), and University of California, Los Angeles/Miller Children’s and Women’s Hospital (Long Beach, CA). Cancer survivorship specialists at each site include oncologists, advanced practice providers, and research/case coordinators (institutional characteristics are given in Table 1).

**Table 1:** Institutional characteristics CHOC, Children's Hospital of Orange County; COH, City of Hope; CHLA, Children's Hospital Los Angeles; KP, Kaiser Permanente Medical Center; RCHSD, University of California, San Diego/Rady Children's Hospital San Diego; UCLA/Miller, University of California, Los Angeles/Miller Children's and Women's Hospital; Cedars, Cedars-Sinai Medical Center; N/A, not applicable

Comprehensive Long-Term Survivorship Program							
Type of institution	CHOC	COH	CHLA	KP	RCHSD	UCLA/Miller	Cedars
Children’s hospital	X		X		X	X	
Cancer centers or university hospital		X	X		X		
Pediatric hospital/unit with adult hospital						X	X
Other				X			
Survivorship program clinical characteristics							
Upper age limit < 21 years			X		X	X	
Upper age limit ≤ 26 years	X						X
No upper age limit		X		X			
When are patients referred? How many years off therapy?	4-5	2	1-2	1-2	2	1-2	
How often are they seen in a disease-specific clinic?	Annual	Annual	Annual	Annual	Every 2 years	Annual	Annual
Dedicated childhood cancer survivorship clinic	X	X	X	X	X	X	
Provide comprehensive treatment summaries	X	X	X	X	X	X	X
Multidisciplinary clinic for childhood cancer survivors	X	X	X	X	X	X	
Dedicated advanced practice practitioner	X	X	X	X	X	X	
Formal transition program to adult survivorship program		N/A	X		X		X

Since its inception, SC-PACS members have met semi-annually in person (until the COVID-19 pandemic), supplemented by monthly teleconferences. Each site’s institutional characteristics, clinical service, and research capabilities were outlined initially. Due to its geographically central location, Children’s Hospital of Orange County functions as the consortium’s administrative hub and governing site. A Data Use Agreement was finalized in February 2018.

We conducted a multi-center, cross-sectional, survey-based study of CACSs and their PPCGs. The goal was to enroll 10 CACSs and 10 PPCGs from each of the seven institutions (total of 140 participants). The eligibility criteria for CACSs were age ≥13 years at the time of enrollment, >2 years from the end of treatment, sufficient cognitive function to complete the survey, and English or Spanish language proficiency. In this cohort, only the CACSs were surveyed. For CACSs < 13 years old, their PPCGs completed the survey. All participants provided written informed consent or assent. The study was approved by each site’s Institutional Review Board (IRB) prior to enrollment of its first participant (Children's Hospital of Orange County In-House IRB #1709102).

Participants were approached during a clinic visit and given a paper questionnaire to complete prior to discharge. The questionnaire was intentionally created for the purpose of this study and was not adapted from a previous, validated tool. Survey items included demographics and 33 questions evaluating the importance and usefulness of health information gained during their survivorship clinic encounters. Feasibility was defined as enrollment of ≥80% of participants who were approached at each site. This was a convenience sample using frequencies and proportions to describe participant characteristics and survey responses, which were entered into a Research Electronic Data Capture (REDCap) database (Vanderbilt University, Nashville, TN).

## Results

Across the consortium, of the recruitment target of 140 participants (CACSs, n=70; PPCGs, n=70), 127 (90.7%) participants were enrolled. At four sites, 20 participants were approached and enrolled; at two sites, 19 and 6 participants, respectively, were approached and enrolled; and at one site, 22 participants were approached and enrolled. A total of 65 (51.2%) participants were CACSs and 62 (48.8%) were PPCGs.

The majority of participants were female (51.2%), were Hispanic (62.2%), spoke English as the primary language at home (57.5%), and were diagnosed between one and four years of age (45.7%) (survivor characteristics are given in Table 2).

**Table 2:** Survivor characteristics

Characteristics	Levels	n (%)
Gender	Female	65 (51.2)
	Male	62 (48.8)
Ethnicity	Hispanic or Latino	79 (62.2)
	Not Hispanic or Latino	44 (34.6)
	Missing responses	3 (2.4)
	I would rather not say	1 (0.8)
Race	White	56 (44.1)
	Asian or Pacific Islander	8 (6.3)
	Black	3 (2.4)
	American Indian/Alaskan Native	3 (2.4)
	Other Asian or Pacific Islander	11 (8.7)
	Missing responses	28 (22.0)
	Multiple responses	9 (7.1)
	I would rather not say	9 (7.1)
Age at diagnosis	<1 year of age	13 (10.2)
	1 to 4 years of age	58 (45.7)
	5 to 9 years of age	32 (25.2)
	10 to 14 years of age	12 (9.4)
	15 to 19 years of age	12 (9.4)
Cancer diagnosis	Leukemia	63 (49.6)
	Lymphoma	16 (12.6)
	Wilms tumor	11 (8.7)
	Bone tumor	6 (4.7)
	Brain tumor	5 (3.9)
	Neuroblastoma	5 (3.9)
	Germ cell tumor	4 (3.1)
	Soft tissue sarcoma	3 (2.4)
	Other (describe)	12 (9.4)
	I do not know	1 (0.8)
	Missing responses	1 (0.8)
Primary language spoken at home	English	73 (57.5)
	Spanish	29 (22.8)
	Arabic	1 (0.8)
	Mandarin/Cantonese	1 (0.8)
	Farsi	1 (0.8)
	Missing responses	3 (2.4)
	Multiple responses	19 (15.0)
Education	Still studying (in elementary, middle, junior/high school)	80 (63.0)
	High school diploma	29 (22.8)
	Associates degree	5 (3.9)
	Bachelor’s degree	4 (3.1)
	Another degree/certificate	3 (2.4)
	Missing responses	4 (3.1)
	I would rather not say	2 (1.6)

Leukemia (63%) and lymphoma (16%) were the most common diagnoses. Information considered most important by both CACSs and PPCGs was related to cancer diagnosis (90.8%) and future risks of treatment (98.0%). There was a slight preference for electronic method of delivery of medical information over paper for both CACSs (38.5% vs. 35.4%) and PPCGs (43.5% vs. 33.9%). Access and resources for psychosocial support were also deemed important by both groups (survey responses are given in Table 3).

**Table 3:** Survey responses *Completed survey for survivors < 13 years old. CACS, childhood/adolescent cancer survivors; PPCG, parents/primary care givers

Variables	Levels	CACS n (%)	PPCG* n (%)
Information about cancer, its treatment, and future health risks	Very important	59 (90.8)	61 (98.4)
	Somewhat important	5 (7.7)	0 (0.0)
	I would rather not say	1 (1.5)	0 (0.0)
	Missing responses	0 (0.0)	1 (1.6)
Information about getting a second cancer in the future	Very important	54 (83.1)	61 (98.4)
	Somewhat important	8 (12.3)	0 (0.0)
	No opinion	1 (1.5)	1 (1.6)
	I would rather not say	2 (3.1)	0 (0.0)
Information about how to check for symptoms that cancer recurs	Very important	57 (87.7)	62 (100.0)
	Somewhat important	7 (10.8)	0 (0.0)
	No opinion	1 (1.5)	0 (0.0)
Concern about current health	Very important	41 (63.1)	56 (90.3)
	Somewhat important	14 (21.5)	3 (4.8)
	Not important	7 (10.8)	1 (1.6)
	No opinion	2 (3.1)	2 (3.2)
	I would rather not say	1 (1.5)	0 (0.0)
Information on how to stay healthy	Very important	47 (72.3)	61 (98.4)
	Somewhat important	12 (18.5)	1 (1.6)
	Not important	5 (7.7)	0 (0.0)
	No opinion	1 (1.5)	0 (0.0)
Information on the proper use and management of medications	Very important	46 (70.8)	52 (83.9)
	Somewhat important	7 (10.8)	4 (6.5)
	Not important	7 (10.8)	4 (6.5)
	No opinion	5 (7.7)	2 (3.2)
Information on having children in the future	Very important	50 (76.9)	57 (91.9)
	Somewhat important	8 (12.3)	4 (6.5)
	Not important	5 (7.7)	0 (0.0)
	No opinion	2 (3.1)	1 (1.6)
Information on health insurance coverage	Very important	47 (72.3)	52 (83.9)
	Somewhat important	12 (18.5)	5 (8.1)
	Not important	4 (6.2)	5 (8.1)
	I would rather not say	2 (3.1)	0 (0.0)
Assistance with healthcare bills and/or health insurance	Very important	35 (53.8)	47 (75.8)
	Somewhat important	16 (24.6)	4 (6.5)
	Not important	8 (12.3)	10 (16.1)
	No opinion	4 (6.2)	1 (1.6)
	I would rather not say	2 (3.1)	0 (0.0)
Information on how the healthcare system works	Very important	47 (72.3)	42 (67.7)
	Somewhat important	12 (18.5)	12 (19.4)
	Not important	4 (6.2)	6 (9.7)
	No opinion	0 (0.0)	2 (3.2)
	I would rather not say	2 (3.1)	0 (0.0)
Access to internet sites to learn more about survivorship	Very important	32 (49.2)	48 (77.4)
	Somewhat important	24 (36.9)	12 (19.4)
	Not important	4 (6.2)	1 (1.6)
	No opinion	5 (7.7)	0 (0.0)
	Missing response	0 (0.0)	1 (1.6)
Access to peer or survivor support groups	Very important	22 (33.8)	21 (33.9)
	Somewhat important	19 (29.2)	36 (58.1)
	Not important	15 (23.1)	4 (6.5)
	No opinion	9 (13.8)	1 (1.6)
Access to camps or other retreats for cancer survivors	Very important	26 (40.0)	36 (58.1)
	Somewhat important	13 (20.0)	19 (30.6)
	Not important	18 (27.7)	6 (9.7)
	No opinion	8 (12.3)	1 (1.6)
Information on where to seek help for managing feelings and learning coping strategies	Very important	26 (40.0)	43 (69.4)
	Somewhat important	19 (29.2)	14 (22.6)
	Not important	14 (21.5)	4 (6.5)
	No opinion	5 (7.7)	0 (0.0)
	Missing responses	1 (1.5)	1 (1.6)
Access to practical support (scholarships, jobs, transportation, etc.)	Very important	43 (66.2)	46 (74.2)
	Somewhat important	16 (24.6)	10 (16.1)
	Not important	6 (9.2)	4 (6.5)
	Missing responses	0 (0.0)	2 (3.2)
Making sure care is coordinated with primary doctor/other providers	Very important	55 (84.6)	56 (90.3)
	Somewhat important	9 (13.8)	3 (4.8)
	Not important	0 (0.0)	2 (3.2)
	I would rather not say	1 (1.5)	0 (0.0)
	Missing responses	0 (0.0)	1 (1.6)
How to talk with non-cancer doctors about cancer and treatment	Very important	35 (53.8)	42 (67.7)
	Somewhat important	18 (27.7)	12 (19.4)
	Not important	4 (6.2)	3 (4.8)
	No opinion	7 (10.8)	1 (1.6)
	I would rather not say	1 (1.5)	2 (3.2)
	Missing responses	0 (0.0)	2 (3.2)
Learning to talk with other people about my cancer and its treatment	Very important	29 (44.6)	37 (59.7)
	Somewhat important	13 (20.0)	11 (17.7)
	Not important	15 (23.1)	10 (16.1)
	No opinion	7 (10.8)	3 (4.8)
	I would rather not say	1 (1.5)	0 (0.0)
	Missing responses	0 (0.0)	1 (1.6)
Information about and access to complementary healthcare	Very important	28 (43.1)	32 (51.6)
	Somewhat important	20 (30.8)	19 (30.6)
	Not important	10 (15.4)	7 (11.3)
	No opinion	6 (9.2)	3 (4.8)
	I would rather not say	1 (1.5)	1 (1.6)
Learning about ways to help other patients or families	Very important	36 (55.4)	46 (74.2)
	Somewhat important	21 (32.3)	11 (17.7)
	Not important	3 (4.6)	3 (4.8)
	No opinion	5 (7.7)	2 (3.2)
Information regarding survivorship that was given during your previous survivorship clinic visit(s)?	Yes, a lot	19 (29.2)	32 (51.6)
	Yes, a little	31 (47.7)	13 (21.0)
	I did not receive any info	8 (12.3)	11 (17.7)
	No, not at all	5 (7.7)	3 (4.8)
	I would rather not say	2 (3.1)	1 (1.6)
	Missing responses	0 (0.0)	2 (3.2)
How useful did you find the survivorship information given to you?	Very useful	27 (41.5)	35 (56.5)
	Somewhat useful	24 (36.9)	13 (21.0)
	Not useful	1 (1.5)	0 (0.0)
	No opinion	2 (3.1)	3 (4.8)
	I would rather not say	1 (1.5)	1 (1.6)
	Missing responses	10 (15.4)	10 (16.1)
How do you prefer to receive information about your survivorship?	Electronic form (e-mail, website)	25 (38.5)	27 (43.5)
	Paper form	23 (35.4)	21 (33.9)
	Mobile app	6 (9.2)	3 (4.8)
	I do not want information	2 (3.1)	0 (0.0)
	I would rather not say	2 (3.1)	1 (1.6)
	Missing responses	7 (10.8)	10 (16.1)
How often do you miss an appointment in the survivorship clinic?	Never	46 (70.8)	36 (58.1)
	Rarely	9 (13.8)	13 (21.0)
	Sometimes	4 (6.2)	7 (11.3)
	Often	4 (6.2)	2 (3.2)
	I would rather not say	1 (1.5)	1 (1.6)
	Missing responses	1 (1.5)	3 (4.8)
Information about my cancer and how it was treated	I am getting the right amount of information	56 (86.2)	50 (80.6)
	I could use more info	9 (13.8)	10 (16.1)
	I am getting too much info	0 (0.0)	2 (3.2)
Information about the healthcare system and how to talk with non-cancer doctors about my cancer diagnosis and its treatment	I am getting the right amount of information	45 (69.2)	39 (62.9)
	I could use more info	17 (26.2)	18 (29.0)
	I am getting too much info	2 (3.1)	2 (3.2)
	I would rather not say	1 (1.5)	2 (3.2)
Information about health conditions resulting from my cancer treatment (such as pain, joint disease, heart disease, development of second cancer, and neurocognitive changes such as decrease memory skills, attention, math skills)	I am getting the right amount of information	45 (69.2)	41 (66.1)
	I could use more info	17 (26.2)	18 (29.0)
	I am getting too much info	2 (3.1)	3 (4.8)
	I would rather not say	1 (1.5)	0 (0.0)
Information or access to complimentary healthcare (such as the use of herbs or vitamins, the practice of yoga or meditation, or receiving chiropractic or acupuncture treatment)	I am getting the right amount of information	41 (63.1)	26 (41.9)
	I could use more info	21 (32.3)	29 (46.8)
	I would rather not say	1 (1.5)	5 (8.1)
	Missing responses	2 (3.1)	2 (3.2)
Learning about ways that I can help other patients or families	I am getting the right amount of information	26 (40.0)	24 (38.7)
	I could use more info	31 (47.7)	30 (48.4)
	I am getting too much info	1 (1.5)	2 (3.2)
	I would rather not say	6 (9.2)	4 (6.5)
	Missing responses	1 (1.5)	2 (3.2)

Overall, there were high levels of satisfaction with the information the survivorship program provided to both group of participants. Overall, 78% of CACSs and PPCGs found the survivorship information useful, and 83% felt that they received the right amount of information about their cancer. The greatest difference between the two groups was the degree of importance for psychosocial support. PPCGs valued access to peer or survivor support groups, and information on where to seek help for managing feelings and learning coping strategies, 29% and 22.8% more, respectively, compared to the CACSs.

## Discussion

The SC-PACS consortium is a unique collaboration of institutions representing varied models of healthcare, including freestanding children’s hospitals, comprehensive cancer centers, managed care organizations, and private institutions. This heterogeneity leverages the strengths of each model, allows testing of interventions in varied settings, and ensures generalizability of results. The ultimate goal of the consortium is to develop a comprehensive survivorship care approach that addresses the most important needs of cancer survivors in our catchment area and promotes best practice interventions.

The demographic reach of the SC-PACS consortium consists of 62% self-reported Hispanic/Latino and 8% Asian. Because of the remarkable racial, ethnic, and sociodemographic diversity of southern California, the SC-PACS consortium stands uniquely in its position for serving and studying the minority population of cancer survivors. We recognize that given this diversity, it may be necessary to expand eligibility criteria of future study participants to patients who are neither completely proficient in English or Spanish and provide questionnaires that can be translated to other languages to promote participation.

Given that this was a feasibility study, our team acknowledged that our small sample size could be insufficient to show statistical significance. Although participants were recruited if they met the eligibility criteria for the study, purposeful selection methods were not rigorously adhered to, thus opening the possibility of selection bias. It is possible that we reached out to CACSs and PPCGs who would be willing to participate and potentially give a more positive feedback.

The importance of building a partnership with other nearby pediatric cancer survivorship centers has been recognized to promote collaborations for research endeavors and educational forums. Another pediatric cancer survivor consortium is the Consortium for New England Childhood Cancer Survivors (CONNECCS). This group was formed in 2011, consisting of 12 academic pediatric oncology institutions, serving a predominantly non-Hispanic white population in the New England region. The successful inception and publications of CONNECCS helped identify challenges and potential strategies for smaller, developing consortia [16-17].

The development of a Consortium Membership Agreement detailing core elements such as membership, data use, and administrative functions was integral to solidifying the consortium, as was establishing a core coordinating center. Moreover, successful completion of our pilot study demonstrates the commitment and ability of member institutions to execute multi-center survivorship studies. Not unexpectedly, some sites encountered administrative delays in obtaining IRB approval, which resulted in two sites being unable to approach 20 participants during the study period. Importantly, however, at both these sites, 100% of the CACSs or PPCGs who were approached did participate. Moving forward, the consortium intends to utilize a central IRB to reduce regulatory burden and facilitate study activation at member sites. Future plans are to expand the needs assessment survey in order to obtain broader representation of the survivor population at SC-PACS institutions. This may, in turn, inform strategies to improve cancer-specific education, delivery of treatment summary, and access to community resources including psychosocial needs for this demographically and socioeconomically diverse population.

## Conclusions

Collaborations with nearby southern California pediatric cancer survivorship centers have enabled us to actively promote research endeavors and educational forums. Through the needs assessment study, we obtained data that specifically characterized our CACS population and their multiple survivorship needs. Future plans are to expand the needs assessment survey in order to obtain broader representation of the survivor population at SC-PACS institutions. This may, in turn, inform strategies to improve cancer-specific education, delivery of treatment summary, and access to community resources including psychosocial needs for this demographically and socioeconomically diverse population.
